# T199. DEVIANT CORTICAL SULCATION RELATED TO SCHIZOPHRENIA, BUT NOT COGNITIVE DEFICITS, LIKELY PREDATE BRAIN DEVELOPMENT IN THE SECOND TRIMESTER

**DOI:** 10.1093/schbul/sby016.475

**Published:** 2018-04-01

**Authors:** Lena Palaniyappan

**Affiliations:** University of Western Ontario

## Abstract

**Background:**

Gestational disruptions are linked to the risk of schizophrenia; but in most cases, there is a lack of a clear history or observable anomaly indicating that the disruptions are likely to be subtle (Murray et al., 2017). The time-locked development of cortical sulci in a human embryo is highly sensitive to developmental disruptions (Chi et al., 1977). We can retrospectively infer the likely timing of embryonic/fetal disruption in schizophrenia by studying the structure of major cortical sulci that represent lobar development in adults with schizophrenia.

**Methods:**

Anatomical T1 MRI scans from a publicly available dataset (COBRE) of 68 patients with schizophrenia and 72 controls were used to evaluate the sulcal depth. 5 major primary sulci that are invariable, representing lobar development (calcarine sulcus, superior temporal sulcus, superior frontal sulcus, interparietal sulcus and inferior frontal sulcus) with formation representing distinct developmental periods (16, 23, 25, 26 and 28 weeks respectively (Chi et al., 1977)) were chosen. Sulcal depth was measured using Morphologist interface of BrainVISA 4.5 (http://brainvisa.info/). Following the construction of 3-dimensional models of cortical folds, various sulci were automatically classified using a probabilistic algorithm with maximum depth computed for each identified sulcus. The 5 sulci were consistently labeled automatically across all subjects. The identified sulci were visually inspected to ensure that the boundaries are in accordance with Ono’s Atlas of Cerebral Sulci (Ono et al., 1990).

**Results:**

A repeated measure ANOVA with 5 sulci and 2 hemispheres as within-subject factors and gender, age and intracranial volume as covariates revealed a significant between-subjects effect for diagnosis (F[1,134]=14.8, p=0.0002). Gender (F[1,134]=7.4, p=0.007) and age (F[1,134]=4.5, p=0.035) also had significant effect in the model. Parameter estimates revealed a significant effect of diagnosis (Controls>Patients) for left superior temporal (t=3.2, p=0.002), right superior temporal (t=2.8, p=0.006), right inferior frontal (t=2.7, p=0.007) and left calcarine (t=2.2, p=0.03) sulci. 5 non-collinear factors representing the 5 bilateral sulci were obtained using varimax rotation, and related to overall MATRICS standardized composite score in patients using multiple regression. The depth of the superior frontal sulcus was the only predictor of the variation in the cognitive score (F[1,54]=8.7, p=0.005).

**Discussion:**

The above findings suggest that the gestational cortical disruption underlying schizophrenia is likely to predate, if not, coincide with the appearance of calcarine sulcus (i.e. 16 weeks, early second trimester) and affects frontal, temporal and occipital lobes. Nevertheless, the burden of cognitive deficits may relate specifically to aberrant superior frontal development occurring in late second trimester.

